# "All Sorts and Conditions" of Women

**Published:** 1889-02-02

**Authors:** 


### The Doctor's Pulpit.

### "ALL SORTS AND CONDITIONS" OF WOMEN.

### The Best Wife.

What standards of comparison ought to be adopted in order to discover the best wife? Are there any judges competent to determine so difficult a practical question? And if there are, what sex do they belong to? Are men fittest to judge of the wifely worth of women, or should other women be placed on the seat of judgment? If by any possibility there should be a day of examination and decision, as the result of which every woman should for the rest of her life occupy first, second, tenth, or ten thousandth rank as a wife, we cannot help wondering whether women would prefer such an examination and decision to be made by a jury of husbands, or by a jury of their fellow-women. The question is crucial. The present writer believes that women themselves would rather be judged by men than by women. If that view be true to fact, women unconsciously admit the intellectual superiority of men. For of all the work to which the human mind may be put, none demands higher, perhaps none demands such high intellectual capacity as that of wise and honest judgment. Failing a jury of husbands, or even of women, to sit upon so unique and vital a question, the writer desires to offer a few hints and suggestions of his own.

Some women, it may be feared, will resent the idea that it can be at all desirable for them to try to belong to the first rank of wives. Their husbands, they will say, and probably with truth, by no means belong to the first rank of husbands. Some of them are meanly selfish; some are exacting to a degree; some are weak in will and irritable in temper, and keep the house in a perpetual state of worry; some are so pleased and satisfied with themselves, that whatever is done for them they take as a matter of course, and as the just recognition of their superlative merits; some are so cold, harsh, and unsympathetic that love and gentleness are as utterly thrown away upon them as if they were lavished on a stone statue. Most true. "And, therefore," say certain of the wives of such husbands, "why should we be everlastingly denying ourselves, constantly repressing our real feelings, and wearing pleasant faces for such bears and brutes as these?" Why, indeed, except that it may be both right and wise to do so? It is both right and wise for every married woman in the world to wish and try to be the best wife in the world.

Now let us here discuss for a moment an important principle of general morality. We will put it in the form of a question: Should a man always strive to do that which is absolutely right, or should he lower his standard to the level of those that are round about him at the time? A moment's consideration will show that the only standard of right which is worthy of the name is that of doing, or striving to do, absolutely right at all times, and under all circumstances. For, consider—a man may suddenly find himself in the midst of cheats and thieves; is he, therefore, to become a cheat and a thief? Or he may find himself surrounded on all hands by liars; must he, therefore, take to lying? Or, being a soldier, he may find most of his companions cravens and cowards; is he then to turn his back upon the enemy and run away with the fleetest? The thing is absurd. It is clear that as a matter of general duty and morality, the only course open to a right-minded man is do right at all times, whether those round about him do right or wrong. This applies with the fullest possible force to the wives of bad or indifferent husbands. If it were desirable to argue about the duty of doing right under any one peculiar circumstance, it would be easy to show why a wife, who must live with her husband whether he is good or not, and who is a mother of children that call the unworthy husband father, should for her children's, her husband's, and her own sake, be the best of wives, even to the worst of husbands. No woman, whatever her husband may be, is exempt from the duty and necessity of being a good wife. No circumstances that can possibly happen can justify her in failing of her duty. It may be her duty to refuse to live with her husband, it may even be her duty to put him in prison; but it can never be her duty to do anything against him which is not right in itself.

What are the essential qualifications for the best wife? Must she be handsome and commanding, rich and influential, strong and instructed in mind, a competent manager of a household, a judicious trainer of children, a hostess who makes her husband's house the gathering-place of all that is most pleasant and distinguished in his neighbourhood? She may be all these or she may be none of them, and yet may be in the truest sense "the best wife." The only two really indispensable qualifications for this honourable distinction are "love and loyalty." The majority of women, we do not hesitate to say, are by nature loving and loyal. Some of them continue to love when even an unreasoning dog could love no more, and many remain loyal in heart long after they have ceased to love. There are men who expect to be loved who never reflect how difficult and even impossible it is to love that which is in itself unlovely. "We needs must love the highest when we see it," says the Laureate. Is the converse true, and is it a necessity of our nature also to "hate the lowest when we see it"? If it be, how hard must be the struggle of some women to keep their hearts from that bitterest of all hatreds, the hatred which usurps the throne of love! A woman, anxious and resolved to do her duty by her husband though she die in the strife of it, will say with the bitterness of despair that she cannot love her husband as she used to do in the early days of their sweet and trustful affection. It may be admitted that she cannot love him "as she used to do." But that is not the same as saying that she cannot love him at all. She may love him with the love of duty, with the love of pity, with the love of memory, with the love of hope. Thousands of women are reduced to necessities of this kind. But she who is able thus to replace the waning warmth of a first love with the gentler but steadier fire of a love of duty or of divine pity does not thereby sink to a lower domestic level, but so far as her individual self is concerned rises to a higher. It is always a sad funeral when a wife has to bury her early love for her husband in the grave of his unworthiness; but with the best women a love both nobler and more enduring always springs up from the ashes.

The husband and wife form a co-partnery for this world and the next, for material prosperity and for intellectual and moral development as well. It may be that they are so unsuitably matched that natural sympathy between them is entirely wanting. It sometimes happens that neither in worldly affairs, nor in mind, nor on moral questions is there any unity of feeling and judgment. What then is to be done? Must they shut themselves up within themselves, and shut out each other? Must they quarrel and fight? What fools they will be if they do either one or the other. They must set themselves first of all to understand each other, and then to raise up between themselves as many common interests as possible. The wife who desires to be one of the best should strive from the very outset to realise her share of this universal co-partnery. In any partnership of two persons one or the other must have the higher moral standard. In marriage, if it be not the husband it must be the wife. Why should it not be the latter as well as the former? No woman can ever be a best wife unless she founds her conduct on a moral basis. She must have a steadfast purpose of heart to do the utmost that is in her, in every detail of life, for the happiness and prosperity of the home and the co-partnery. If she sets out with this principle, and adheres steadily to it, details of house management, of expenditure, of sympathy and help for her husband, will arrange themselves. In wedded life the husband's care is the health of his finances, the wife's that of domestic health. The moral and intellectual atmosphere of the house must be in the keeping of both. If each does the best that is possible in every department, happiness and prosperity are practically certain. But, in any case, whatever the husband does, "the best wife" will leave nothing undone which it is possible for her to accomplish.

*(To be continued.)*

